# SPSB2 inhibits hepatitis C virus replication by targeting NS5A for ubiquitination and degradation

**DOI:** 10.1371/journal.pone.0219989

**Published:** 2019-07-25

**Authors:** Mingzhen Wang, Yu Wang, Yuehong Liu, Hailong Wang, Xiu Xin, Jiadai Li, Yao Hao, Lingling Han, Fang Yu, Congyi Zheng, Chao Shen

**Affiliations:** 1 State Key Laboratory of Virology, College of Life Sciences, Wuhan University, Wuhan, China; 2 Department of Pathology, Zhongnan Hospital, Wuhan University, Wuhan, China; 3 China Center for Type Culture Collection, Wuhan University, Wuhan, China; Academia Sinica, TAIWAN

## Abstract

Hepatitis C virus (HCV) replication involves many viral and host factors. Host factor SPRY domain- and SOCS box-containing protein 2(SPSB2) belongs to SPSB family, and it recruits target proteins by the SPRY domain and forms E3 ubiquitin ligase complexes by the SOCS box. As an adaptor protein, it can regulate the host’s response to infection, but little is known about whether SPSB2 plays a role in HCV replication. In the present study, we found that HCV infection significantly upregulated the mRNA and protein levels of SPSB2 in HCVcc-infected cells. Exogenous expression of SPSB2 in hepatoma cells decreased HCV RNA and protein levels which depended on the SOCS box, while knockdown of endogenous SPSB2 increased HCV RNA and protein levels. Additionally, we demonstrated that SPSB2 interacted with HCV structural protein E1 and nonstructural protein protein 5A (NS5A) via the C-terminal portion of the SPSB2 SPRY domain. Furthermore, SPSB2 induced NS5A ubiquitination and mediated NS5A degradation. Collectively, this study discovered host factor SPSB2 significantly inhibits HCV replication by interacting and degrading NS5A.

## Introduction

With an estimated 71 million people infected worldwide despite the development of direct-acting antivirals (DAAs), Hepatitis C virus (HCV) remains a considerable threat to global health. Among those with acute HCV infection, 80% develop a persistent infection with a high risk of causing chronic hepatitis, cirrhosis, and hepatocellular carcinoma[1]. Although HCV-infected patients are now treated with DAA therapy rather than the combination of pegylated interferon-α and ribavirin, these patients still face several hurdles, such as high treatment costs, drug-resistant variants due to inherent characteristics of RNA viruses, strong pathogenicity of multiple HCV genotypes, reinfection after treatment, and no preventive HCV vaccine[2,3]. Because HCV infection cycle is tightly associated with viral and host proteins, an alternative strategy to combat HCV is targeting host proteins [4,5].

HCV is a small enveloped virus that belongs to the Flaviviridae family with a positive-stranded RNA genome, which encodes a large polyprotein of 3010 amino acids. The polyprotein is cleaved co- and post-translationally by cellular and viral proteases into structural proteins (core, E1, E2) which build up the viral particles and nonstructural proteins (p7, NS2, NS3, NS4A, NS4B, NS5A, and NS5B) that participate in virus replication and assembly processes[6]. NS5A includes an N-terminal amphipathic helix domain, which is important for associating with the membrane, and three domains (DI–DIII) separated by two linker regions named LCSI and LCSII. Several studies show that DI and DII play vital roles in HCV replication and that DIII participates in virus particle assembly [7–9]. Additionally, previous studies indicate that factors that change NS5A conformation, sub-cellular localization, and protein amount affect the role of NS5A in HCV RNA replication and virus particle assembly [10–14]. The ubiquitination and proteasome degradation of NS5A leads to less NS5A protein, inhibiting HCV replication and virus particle assembly. To date, three host factors have been identified that cause NS5A ubiquitination and proteasome degradation to hinder HCV replication and virus particle assembly: TRIM14, TRIM22, and ISG12a [15–17]. As a multifunctional protein in HCV replication and assembly, NS5A is a significant target for potent antiviral therapy.

The SPRY domain and SOCS (suppressor of cytokine signaling) box-containing proteins,SPSB1-4,are characterized by the central SPRY protein binding domain and a C-terminal SOCS box-containing domain[18]. A SOCS box is approximately 40 residues in length containing a BC box and a Cul5 box, which recruit an adaptor protein (Elongin B/C) and Cullin5, respectively [19]. Cullin5, a scaffold protein, assembles multiple proteins into complexes consisting of a small RING finger protein (Rbx2), Elongin B and C, and the substrate-targeting protein with a SOCS box to form Elongin B/C-Cullin 5-SOCS box protein (ECS) E3 ligase complexes[20]. Together with ubiquitin-activating enzyme (E1) and ubiquitin-conjugating enzyme (E2), the E3 enzyme cascade forms a ubiquitin-proteasome system that regulates various cellular events, such as cell-cycle control and cell proliferation, DNA repair, apoptosis, cellular stress regulation, and immune response by degradation regulatory proteins [21]. Functioning as adaptor proteins in ECS E3 ligase complexes, SPSB proteins are responsible for recruiting target protein by the SPRY domain and forming ECS complexes by the SOCS box, which result in target protein ubiquitination and degradation. SPSB1, SPSB2, and SPSB4 can interact with inducible nitric oxide synthase (iNOS) resulting in its proteasomal degradation to regulate the host’s response to infection [22,23]. SPSB1 negatively regulates TGF-β signaling by interacting with TβRII via its SPRY domain and facilitating the degradation of TβRII, resulting in the inhibition of tumor cells migration and invasion [24]. SPSB3 targets SNAIL for polyubiquitination and proteasomal degradation to suppress tumor metastasis in various kinds of cancer, suggesting the vital role of SPSB3 in regulating epithelial-mesenchymal transition and cancer progression [25]. SPSB4 plays a crucial role in regulating cellular repulsive responses through interacting with and targeting EphB2 for degradation [26].

The roles of SPSB proteins in virus replication remain enigmatic. In the present study, the expression of SPSB proteins induced by HCV infection and their roles in HCV replication were investigated, and the antiviral function and mechanism of SPSB2 was delineated. The results demonstrated that SPSB2 significantly inhibits HCV replication by targeting NS5A for ubiquitination and degradation.

## Materials and methods

### Cell lines

Human hepatoma Huh7 and Huh7.5.1 cells (kindly provided by Francis V. Chisari, Scripps Research Institute) were cultured in Dulbecco’s modified Eagle’s medium supplemented with 10% fetal bovine serum (HyClone), 100 U/ml penicillin, and 100 U/ml streptomycin (Beyotime). Human embryonic kidney (HEK) 293T cells were cultured in minimal essential medium supplemented with the same reagents as Huh7 and Huh7.5.1 cells. All cells were grown at 37°C in a humidified incubator with a 5% CO_2_ atmosphere.

### Plasmids and reagents

Plasmids expressing host factors SPSB1–4 and SPSB2 truncation mutants were constructed into the mammalian expression vector pKH3 using cDNA as a template from Huh7 cells. Viral genes coding HCV proteins were cloned into mammalian expression vectors pEGFP-C1 (Clontech), pRK-7 (Addgene) with a 5′-Flag tag, or pRK-5 (Addgene) with a 5′-HA tag using the template amplified from cDNA of Huh7.5.1 cells infected with HCV JFH-1. pHA-Ub and pMYC-Ub plasmids were kindly provided by Jianguo Wu (College of Life Science, Wuhan University, China). SiNC and SiSPSB2 were purchased from Santa Cruz Biotechnology and dissolved in RNase-free water. Cycloheximide was purchased from Sigma and dissolved in dimethyl sulfoxide. The proteasome inhibitor MG132 was purchased from Selleck.

### HCVcc infection

Infectious HCV (genotype 2a, strain JFH-1) was provided by Takaji Wakita (National Institute of Infectious Diseases, Japan); the virus amplification, titer measure, and storage were carried out as described previously [27]. HCV infection was performed as described previously [28]. Briefly, serum-free medium used to dilute JFH-1 was added to Huh7 or Huh7.5.1 cells, the medium was changed after 6 h, and the cells were cultured in fresh medium up to the indicated time points and harvested for RNA and protein expression analysis. For the experiments assessing the effects of host factors and truncation mutant on HCV replication, HCV infection was performed after transfection with the indicated plasmids or siRNAs using Lipofectamine 2000 reagent (Invitrogen) according to the manufacturer’s instructions.

### RNA extraction and quantitative RT-PCR

Total RNA from cultured cells was extracted with TRIzol reagent (Invitrogen) according to the manufacturer’s instructions. Then, cDNA was synthesized from 1 μg total RNA in a 20-μl volume using Moloney murine leukemia virus reverse transcriptase (Promega) and an N6 random primer (Bioneer). The qRT-PCR experiment was conducted using a CFX96 real-time PCR detection system (Bio-Rad) with SYBR green dye (Invitrogen). Relative intracellular HCV RNA levels and other cellular genes RNA levels were determined by normalization to GAPDH RNA levels. The following qRT-PCR primers used for gene detection: FP (GAPDH), CCACTCCTCCACCTTTGAC; RP (GAPDH), ACCCTGTTGCTGTAGCCA; FP (JFH-1), TCTGCGGAACCGGTGAGTA; RP (JFH-1), TCAGGCAGTACCACAAGGC; FP (SPSB1), AAGCTCATCTTTCACCGGCA; RP (SPSB1), GGGTTGTGTACCCGACAGAG; FP (SPSB2), TGGACATGGAGGAGGGAACT; RP (SPSB2), TAGAGGGTCCTGCCCTTCAG; FP (SPSB3), ACTGGGGCTACGACTCTGAT; RP (SPSB3), GAGTGCAGGCTGCTACAGAA; FP (SPSB4), CTTCATCGTGGATGGCCAGTA; and RP (SPSB4), GGATCTCCGGCACAGGTC.

### Co-immunoprecipitation and western blotting

For co-immunoprecipitation experiments in HEK293T cells, the indicated plasmids were transfected into HEK293T cells seeded in 10-cm dishes with calcium phosphate. The transfected cells were cultured for 36 hours, then washed with ice-cold PBS three times, and lysed in 1 ml western and immunoprecipitation lysis buffer (Beyotime) containing cocktail (Roche) for 20 min on ice. The lysates were collected and centrifuged at 13000 g for 15 min at 4°C. The supernatants at equal volume were incubated with the indicated antibody or control immunoglobin G (IgG) for 2 h, and then incubated with 50 μl protein A/G agarose (Beyotime) for 2 h. The beads were collected by centrifugation at 5000 rpm for 30 s at 4°C and then washed three times with a buffer containing 15 mM Tris (pH 7.5), 500 mM NaCl, 2 mM EDTA, and 0.5% Triton X-100. The bound proteins were boiled in 2X SDS loading buffer for 10 min and subjected to western blotting. For the endogenous co-immunoprecipitation assay, Huh7.5.1 cells infected with JFH-1 for 96 h were lysed and subjected to the procedure described above.

For western blotting experiments, samples were subjected to SDS-PAGE, transferred onto polyvinylidene difluoride membrane (Bio-Rad), and then blocked with 5% skimmed milk in Tris-buffered saline with 0.05% Tween 20. Blots were incubated with the indicated primary antibodies, followed by a secondary antibody conjugated to horseradish peroxidase (HRP). Imaging was performed using an enhanced chemiluminescence reagent (Millipore). The following primary and second antibodies were used: mouse anti-actin (1:5000) (Proteintech); mouse anti-HCV core (1:500) (Santa Cruz Biotechnology); mouse anti-HCV NS5A(1:2000) (BioFront); mouse anti-SPSB2 (1:500) (Santa Cruz Biotechnology); rabbit anti-Flag (1:3000) (Proteintech); mouse anti-Flag (1:1000) (Beyotime); mouse anti-HA (1:2000) (Medical Biological Laboratories); rabbit anti-Myc (1:3500) (Proteintech); mouse anti-GFP(1:2000) (Proteintech); anti-mouse IgG-HRP secondary antibody (1:10000) (Proteintech); and anti-rabbit IgG-HRP secondary antibody (1:10000) (Proteintech).

### Immunofluorescence staining and confocal microscopy

Huh7 cells were cultured on a 20-mm glass bottom dish (NEST) and transfected with the indicated plasmids. Then the cells were washed three times with PBS, fixed with 4% paraformaldehyde–containing PBS for 20 min, and permeabilized with 1% Triton X-100–containing PBS for 20 min. After being blocked with 2% bovine serum albumin–PBS for 1 h, the cells were incubated with primary antibody diluted in antibody dilution buffer overnight at 4°C.After being washed three times with PBS, the cells were incubated with second antibody for 1 h and washed with PBS for three times. Then the cells were treated with DAPI (Beyotime) for 10 min, washed three times with PBS, and observed with a confocal microscope (Olympus). The following primary and second antibodies were used: mouse anti-NS5A antibody 9E10 was kindly provided by Chaoyang Li (Wuhan Institute of Virology, Chinese Academy of Sciences, China); mouse anti-Flag antibody was purchased from Beyotime; rabbit anti-Myc antibody was purchased from Proteintech; TRITC-conjugated or FITC-conjugated second antibodies were purchased from Pierce.

### Statistical analysis

Statistical graphs were generated by GraphPad Prism5 software, and statistical analyses were performed with Student’s t-test. The data presented are representative of at least three independent experiments.

## Results

### HCV upregulates SPSB2 mRNA and protein levels

**Fig 1:**
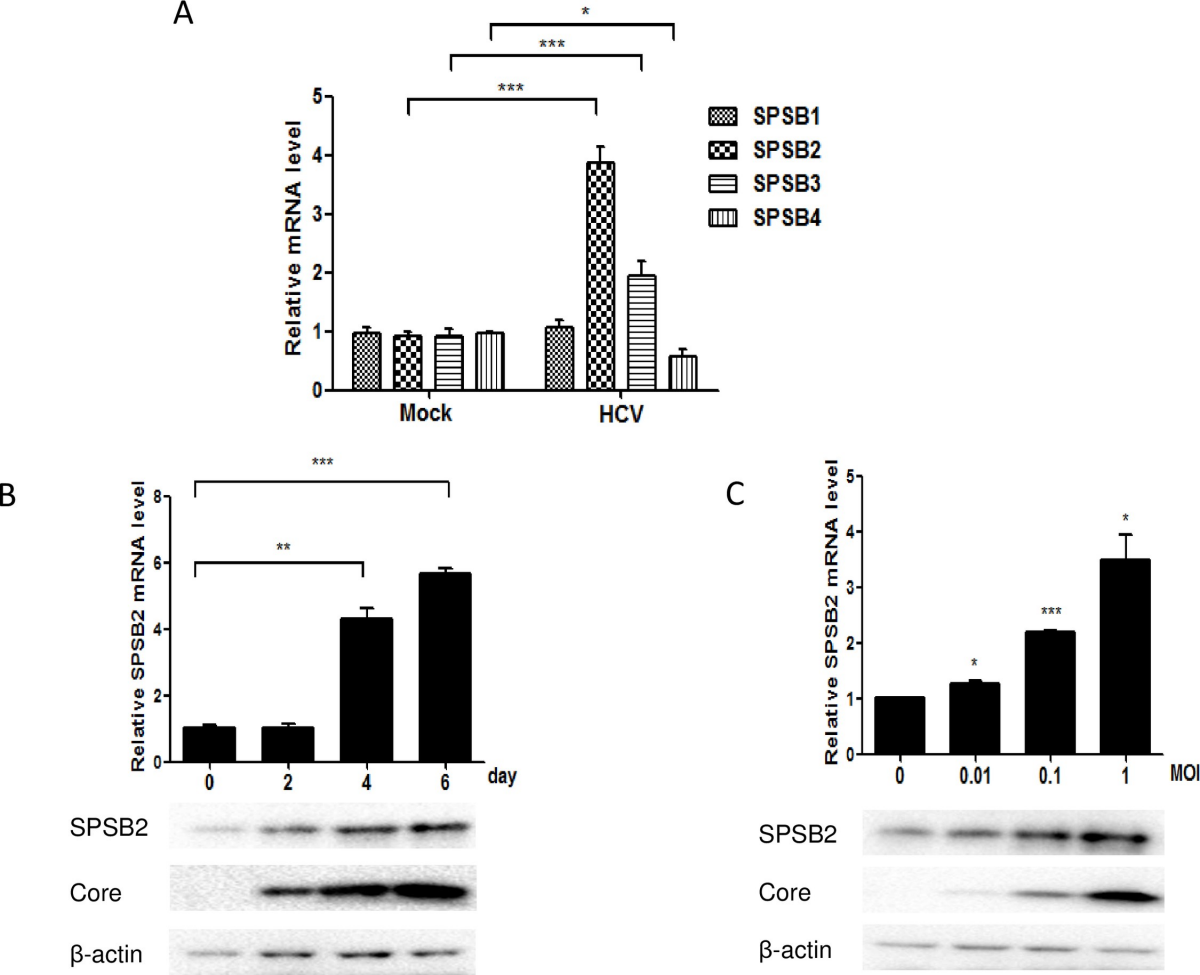
Hepatitis C virus (HCV) infection upregulates SPSB2 mRNA and protein levels. (A) Huh7.5.1 cells were incubated for 6 h with HCV JFH-1 (multiplicity of infection [MOI] = 1) and cultured for another 96 h with fresh medium; the mRNA was quantified by qRT-PCR. (B) Huh7.5.1 cells were infected with JFH-1 (MOI = 1), incubated for 6 h, and harvested at different time points; SPSB2 mRNA and protein were analyzed by qRT-PCR and western blot, respectively. (C) Huh7.5.1 cells were infected with JFH-1 at different virus titers, incubated for 6 h, and harvested for SPSB2 mRNA and protein detection after another 96 h by qRT-PCR and western blot, respectively. Experiments were performed three times with similar results. Data represent the means ± SD (n = 3). *P < 0.05; **P < 0.01; ***P < 0.001.

Previous studies have indicated that HCV infection can modulate the transcription and expression of host genes, both of which influence host survival or virus replication. To investigate the effects of HCV infection on the expression of SPSB family members SPSB1–4, Huh7.5.1 cells were infected with HCV JFH-1 (multiplicity of infection [MOI] = 1), and mRNA levels of SPSB1–4 were determined by qRT-PCR. Compared to the mock-infected cells, the mRNA of SPSB1 in HCV-infected cells was not changed; the mRNA levels were upregulated approximately 3.8 folds for SPSB2 and 1.9 folds for SPSB3. By contrast, SPSB4 mRNA level was downregulated approximately 45% (Fig 1A). HCV RNA level was detected to indicate HCV infection efficiency (S1A Fig). These results showed that the change in SPSB2 mRNA was the most obvious response to HCV infection.

Next, SPSB2 mRNA and protein levels were measured at various time points after infection with JFH-1 (MOI = 1); the results showed that SPSB2 mRNA and protein levels were upregulated 4 and 6 days after infection (Fig 1B). HCV RNA level was shown in S1B Fig. Additionally, the SPSB2 mRNA level and protein levels were detected after HCV infection at different titers compared to that in mock-infected cells; the results demonstrated that upregulation of SPSB2 mRNA and protein levels were positively correlated with HCV infection titers (Fig 1C).HCV RNA level was shown in S1C Fig. Together, these data indicated that HCV positively regulated SPSB2 mRNA and protein expression.

### SPSB2 inhibits HCV replication

**Fig 2:**
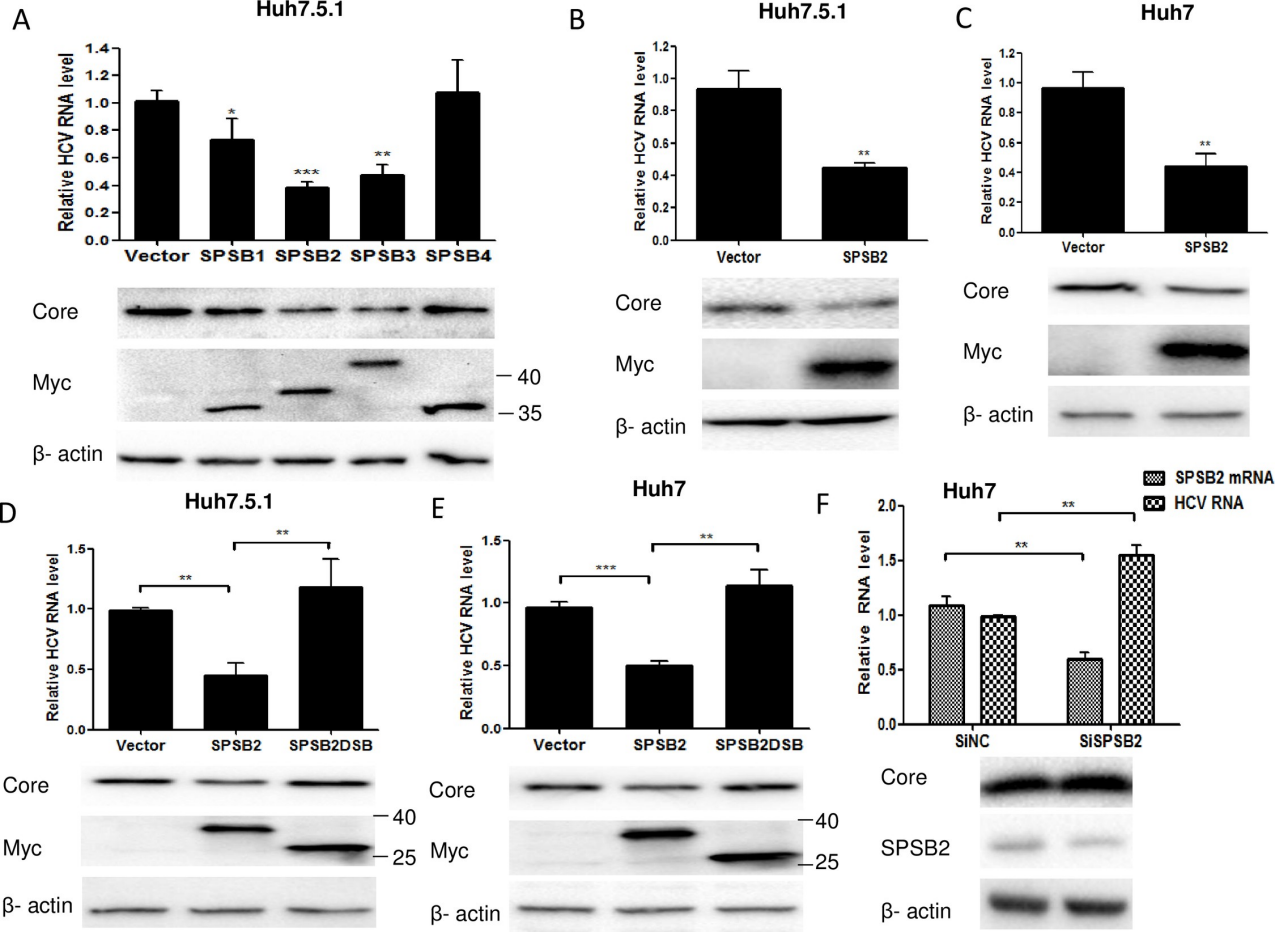
SPSB2 inhibits hepatitis C virus (HCV) replication. (A) Huh7.5.1 cells were transfected with plasmids expressing SPSB1, SPSB2, SPSB3, SPSB4, or an empty vector (Myc tagged) for 24 h and then infected with HCV JFH-1 (multiplicity of infection [MOI] = 0.1) for 72 h; HCV RNA and protein levels were quantified by qRT-PCR (upper) and western blot (lower), respectively. (B) Huh7.5.1 cells were infected with JFH-1 (MOI = 0.1) for 6 h and transfected with plasmids expressing SPSB2 or an empty vector (Myc tagged) for 72 h. HCV RNA was quantified by qRT-PCR (upper), and protein levels were detected by western blot (lower). (C) Huh7 cells were infected with JFH-1 (MOI = 0.1) after being transfected with plasmids expressing SPSB2 or an empty vector (Myc tagged) for 24h; HCV RNA and protein levels were analyzed by qRT-PCR (upper) and western blot (lower) after being cultured for 72 h. (D) Huh7.5.1 cells and (E) Huh7 cells were transfected with plasmids encoding for SPSB2, SPSB2DSB, or an empty vector (Myc tagged) for 24 h and then were infected with JFH-1 (MOI = 0.1) for 72 h. The HCV RNA and protein levels are shown in the upper and lower panels, respectively. (F) Huh7 cells were transfected with SiNC or SiSPSB2 for 24 h and then were infected with JFH-1 (MOI = 0.1) for 72 h. The HCV RNA and protein levels are shown in the upper and lower panels, respectively. Experiments were performed three times with similar results. Data represent the means ± SD (n = 3). *P < 0.05; **P < 0.01; ***P < 0.001.

To understand whether SPSB family members SPSB1–4 were involved in the HCV life cycle, Huh7.5.1 cells transfected with plasmids expressing SPSB1, SPSB2, SPSB3, and SPSB4 were infected with HCV JFH-1 (MOI = 0.1). The qRT-PCR and Western blot results showed that overexpression of SPSB2 and SPSB3 decreased HCV RNA and core protein levels, but overexpression of SPSB1 and SPSB4 had only minimal effects on HCV RNA and core protein levels (Fig 2A). Because it had the greatest effect on HCV replication, SPSB2 was chosen for further investigation.To examine whether changing the sequence of HCV infection and plasmid transfection altered the antiviral effect of SPSB2 on HCV replication, Huh7.5.1 cells were first infected with HCV and then transfected with plasmids expressing SPSB2 or an empty vector. The results showed that SPSB2 overexpression also inhibited HCV RNA and core protein levels (Fig 2B). Additionally, Huh7 cells were infected with HCV after being transfected with plasmids expressing SPSB2 or an empty vector, and a similar antiviral effect of SPSB2 was observed (Fig 2C).Because the SOCS box is important for the function of SPSB2 as a member of SOCS family proteins, the SPSB2 deletion mutant (SPSB2DSB) lacking SOCS box was used to examine its effect on HCV replication. The results showed that SPSB2DSB overexpression disrupted inhibition of HCV replication compared to SPSB2 overexpression in Huh7 and Huh7.5.1 cells (Fig 2D and 2E). In addition, knockdown of endogenous SPSB2 in Huh7 cells increased HCV RNA and protein levels (Fig 2F). Collectively, these data demonstrated that SPSB2 significantly inhibits HCV replication in hepatoma cells, and this effect is dependent on its SOCS box.

### SPSB2 interacts with HCV E1 and NS5A

**Fig 3:**
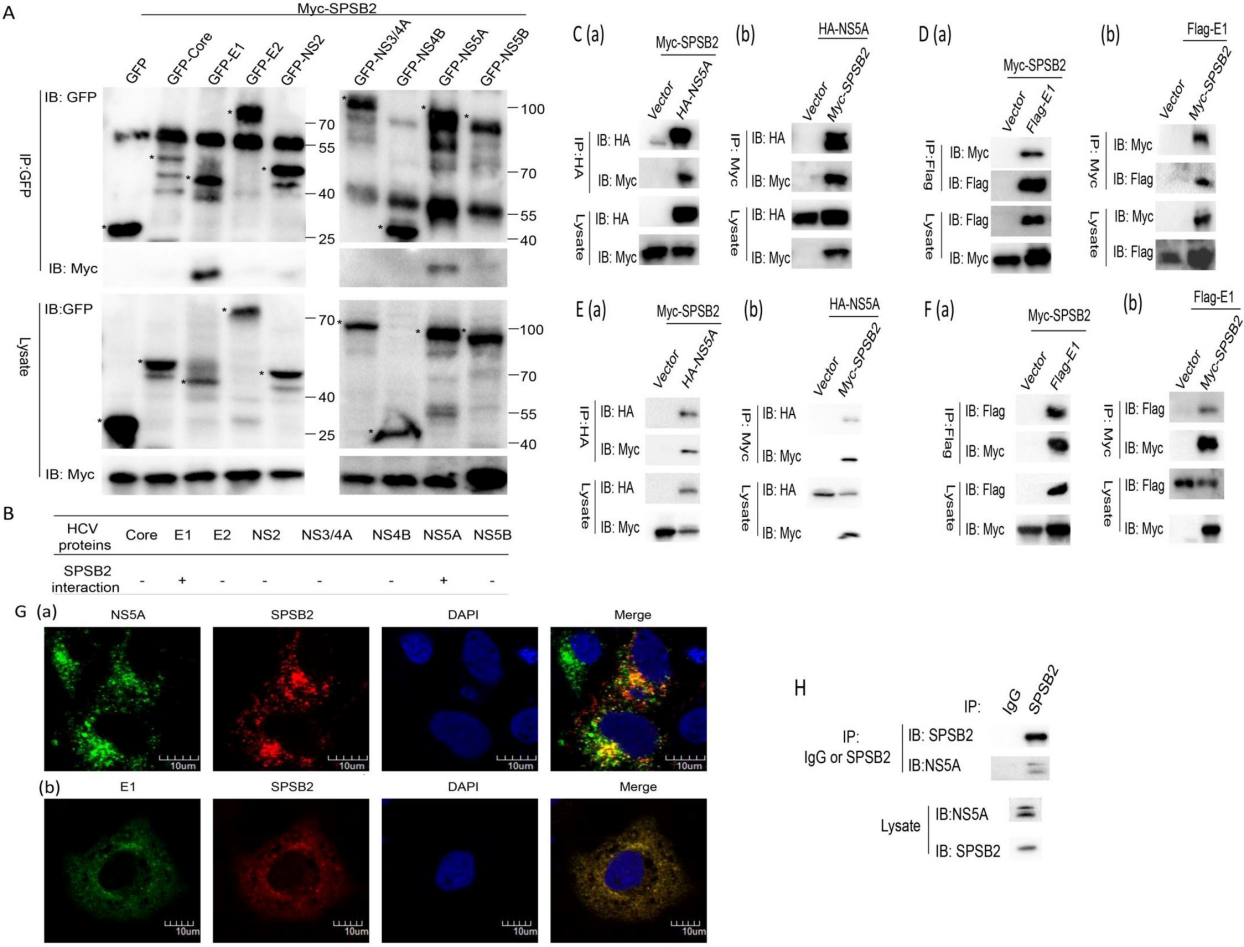
SPSB2 interacts with hepatitis C virus (HCV) E1 and nonstructural protein 5A (NS5A). (A) Myc-SPSB2 was co-transfected with the plasmids coding HCV proteins (core, E1, E2, NS2, NS3/4A, NS4B, NS5A, or NS5B) or pEGFP-C1 (empty vector) for 36 h in HEK293T cells; cell lysates were immunoprecipitated with anti-GFP antibody, and the immunoprecipitates were analyzed by western blot with anti-GFP or anti-Myc antibody. (B) Schematic diagram of the interaction between SPSB2 and HCV proteins. (C) Myc-SPSB2 was co-transfected with HA-NS5A or an empty vector in HEK293T cells for 36 h; cell lysates were immunoprecipitated with anti-HA antibody (a) or anti-Myc antibody (b), and the immunoprecipitates were analyzed by western blot with anti-HA or anti-Myc antibody. (D) Myc-SPSB2 was co-transfected with Flag-E1 or an empty vector in HEK293T cells for 36 h; cell lysates were immunoprecipitated with anti-Flag antibody (a) or anti-Myc antibody (b), and the immunoprecipitates were analyzed by western blot with anti-Flag or anti-Myc antibody. (E) Myc-SPSB2 was co-transfected with HA-NS5A or an empty vector in Huh7 cells for 48 h; cell lysates were immunoprecipitated with anti-HA antibody (a) or anti-Myc antibody (b), and the immunoprecipitates were analyzed by western blot with anti-HA or anti-Myc antibody. (F) Myc-SPSB2 was co-transfected with Flag-E1 or an empty vector in Huh7 cells for 48 h; cell lysates were immunoprecipitated with anti-Flag antibody (a) or anti-Myc antibody (b), and the immunoprecipitates were analyzed by western blot with anti-Flag or anti-Myc antibody. (G) Huh7 cells were transfected with Myc-SPSB2 after infected with HCV JFH-1 (a); Huh7 cells were transfected with Myc-SPSB2 and GFP-E1 (b), and then stained with anti-NS5A antibody 9E10 and FITC-conjugated goat anti-mouse secondary antibody to detect NS5A, as well as anti-Myc antibody and TRITC-conjugated goat anti-rabbit secondary antibody to detect Myc-SPSB2. Cells were stained with DAPI to visualize the nuclei. (H) HCV JFH1-infected Huh7.5.1 cells were harvested after being cultured for 96 h,and lysates were immunoprecipitated with a mouse anti-SPSB2 MAb or control mouse IgG. The immunoprecipitates were subjected to western blot with anti-NS5A (JFH-1) antibody and anti-SPSB2 antibody.

SPSB2, as an adaptor protein, may interact with many host proteins by its SPRY domain. To investigate whether SPSB2 can bind to any HCV proteins, HCV proteins (core, E1, E2, NS2, NS3/4A, NS4B, NS5A and NS5B) tagged with green fluorescent protein (GFP) were co-expressed with SPSB2 in HEK293T cells. Co-immunoprecipitation results showed that SPSB2 interacted with HCV E1 and NS5A (Fig 3A and 3B). To consider the possible influence of GFP on HCV E1 and NS5A conformation, Flag-E1 or HA-NS5A was co-expressed with SPSB2 in HEK293T cells. Co-immunoprecipitation results showed were consistent with those for GFP-fused E1 and NS5A (Fig 3C and 3D). Also, Co-immunoprecipitation assays about the interaction between SPSB2 and HCV proteins E1/NS5A were performed in Huh7 cells, and the results were consistent with those in HEK293T cells (Fig 3E and 3F). To determine the relationship between subcellular localization of SPSB2 and HCV proteins, the immunofluorescence assay was performed in Huh7 cells, the results showed that both E1 and NS5A were colocalized with SPSB2 (Fig 3G).

Next, to investigate whether the interactions occurred in the HCV replication system, JFH-1-infected Huh7.5.1 cell lysates (MOI = 0.1) were subjected to immunoprecipitation analysis by western blotting with anti-SPSB2 antibody. The results showed that endogenous SPSB2 could interact with NS5A in the authentic system (Fig 3H). However, due to lack of HCV JFH-1 E1 antibody, the interaction between SPSB2 and E1 in the HCV infection system could not be further explored.

### The C-terminal portion of SPSB2 SPRY domain is critical for binding with E1 and NS5A

**Fig 4:**
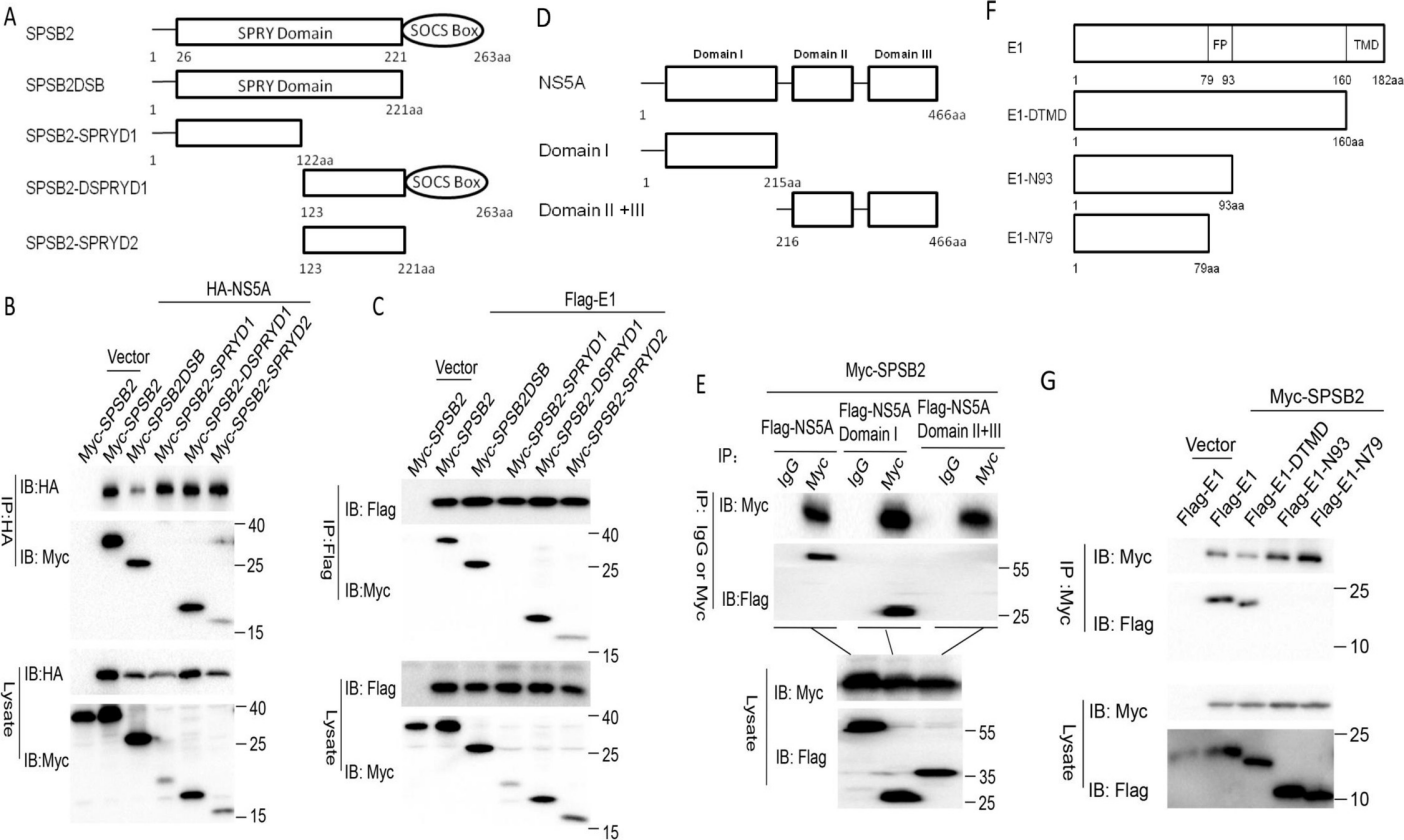
The C-terminal portion of the SPSB2 SPRY domain is critical for binding with E1 and nonstructural protein 5A (NS5A). (A) Schematic diagram of full-length SPSB2 and its mutants. (B) Immunoprecipitation (IP) analysis of NS5A and SPSB2 mutants from HEK293T cells. (C) IP analysis of E1 and SPSB2 mutants from HEK293T cells co-expressing E1 and SPSB2 mutants. (D) Schematic diagram of full-length NS5A and mutants. (E) IP analysis of SPSB2 and NS5A mutants from 293T cells co-expressing SPSB2 and NS5A mutants. (F) Schematic diagram of full-length E1 and its mutants. (G) IP analysis of SPSB2 and E1 mutants from 293T cells co-expressing SPSB2 and E1 mutants.

Next, we wanted to determine which domain of SPSB2 is necessary for its interaction with E1 and NS5A. The major functional domains of SPSB2 are illustrated in Fig 4A. We performed immunoprecipitation analyses with full-length NS5A and various domains of SPSB2. The results showed that the truncation mutants SPSB2DSB (1-221aa), SPSB2-DSPRYD1 (123-263aa), and SPSB2-SPRYD2 (123-221aa) were able to interact with NS5A, while the N-terminal portion SPSB2-SPRYD1 (1-122aa) did not interact with NS5A (Fig 4B). We concluded that SPRYD2 (123-221aa) of SPSB2 was the critical NS5A-binding domain. To investigate which domain of SPSB2 was necessary for interaction with E1, an immunoprecipitation experiment was performed with full-length E1 and various domains of SPSB2. The results indicated that SPRYD2 (123-221aa) of SPSB2 was also the critical domain for binding to E1 (Fig 4C). Meanwhile, immunofluorescence assays were performed to explore the effects of SPRYD2 domain on the localization relationship between SPSB2 and HCV proteins, the results showed that both SPSB2 and SPSB2-DSB were obviously colocalized with E1/NS5A, but no obvious colocalization was observed when SPRYD2 domain was deleted (S2 and S3 Figs).

NS5A has three functional domains (Domain I, Domain II and Domain III) (Fig 4D). We performed immunoprecipitation analyses with full-length SPSB2 and various domains of NS5A. The results showed that both NS5A and NS5A DI (NS5A Domain I) could interact with SPSB2, but NS5ADII+DIII (NS5A Domain II and Domain III) could not interact with SPSB2. These results indicated that Domain I of NS5A was responsible for interaction with SPSB2 (Fig 4E).

E1 has two important domains: one is the fusion peptide (FP) which contributes to HCV entry and virus morphogenesis, and the other is the transmembrane domain (TMD) which is responsible for E1 and E2 to form a stable heterodimeric complex that mediates virus entry. To determine which domain of E1 is responsible for interaction with SPSB2,Co-immunoprecipitation assays were performed with full-length SPSB2 and various domains of E1 (Fig 4F). The results showed that both E1 and E1-DTMD could interact with SPSB2, but E1-N93 and E1-N79 could not interact with SPSB2. These results indicated that 94-160aa of E1 was necessary for interaction with SPSB2 (Fig 4G).

### SPSB2 induces the ubiquitination and degradation of NS5A

**Fig 5:**
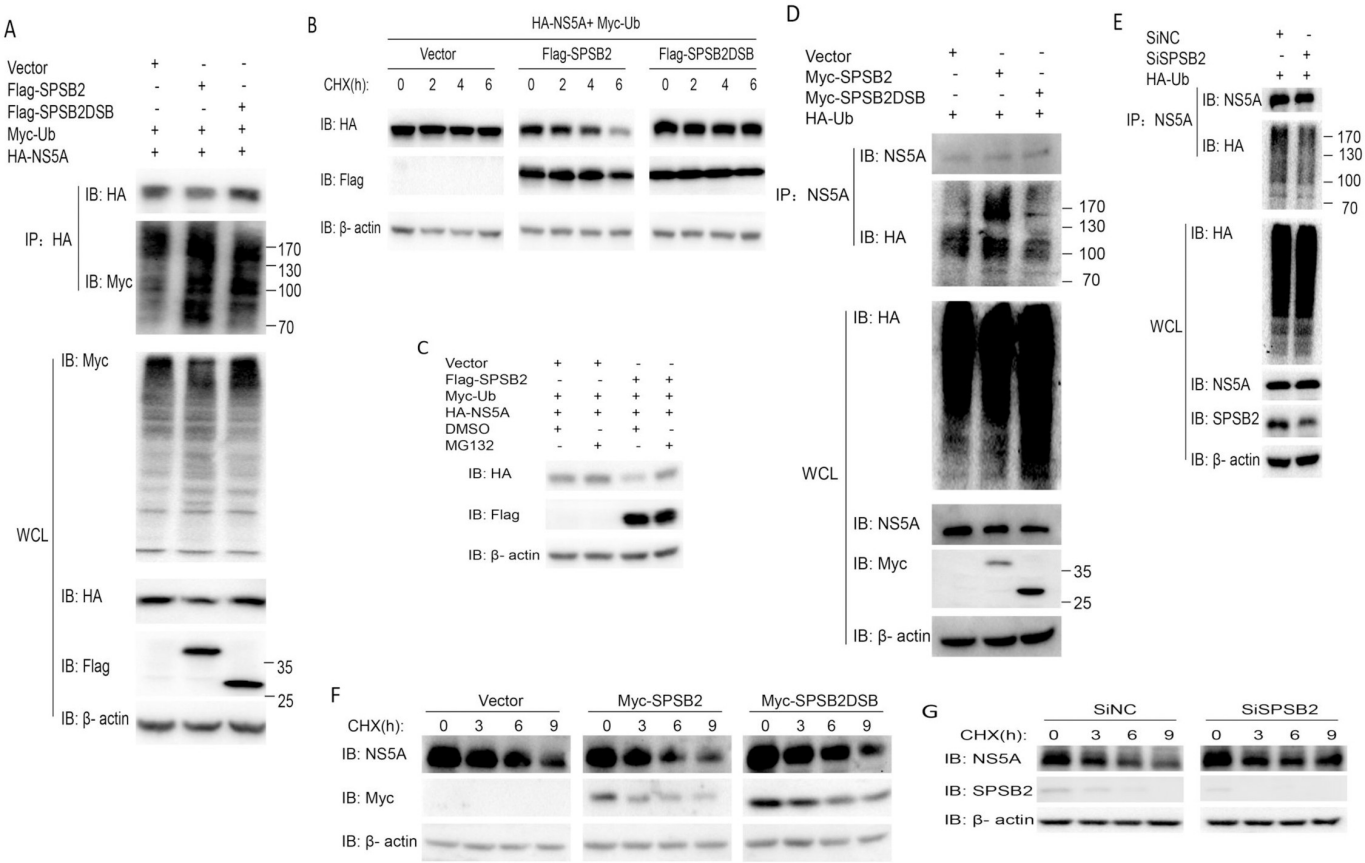
SPSB2 induces the ubiquitination and degradation of nonstructural protein 5A (NS5A). (A) HA-NS5A and Myc-Ub were transfected with Flag-SPSB2, Flag-SPSB2DSB, or an empty vector in HEK293T cells for 48 h; cell lysates were immunoprecipitated with anti-HA antibody. The immunoprecipitates were analyzed for indicated proteins by western blot. (B) 293T cells were co-transfected with various constructs as indicated. After 36 h, cells were exposed to 25 μg/ml cycloheximide (CHX) for the indicated periods. Whole cell lysates were then examined for the indicated proteins by western blot. (C) 293T cells were transfected with the indicated plasmids, and then cells were treated with dimethyl sulfoxide (DMSO) or the proteasome inhibitor MG132 for 6 h. Whole cell lysates were then examined for the indicated proteins by western blot. (D) Huh7 cells were infected with JFH-1 (MOI = 0.1) after transfected with plasmids expressing SPSB2, SPSB2DSB or an empty vector. After 72 h, cells were treated with proteasome inhibitor MG132 for 8 h. cell lysates were immunoprecipitated with anti-NS5A antibody. The immunoprecipitates were analyzed for indicated proteins by western blot. (E) Huh7 cells were infected with JFH-1 (MOI = 0.1) after transfected with SiNC or SiSPSB2. After 72 h, cells were treated with proteasome inhibitor MG132 for 8 h. cell lysates were immunoprecipitated with anti-NS5A antibody. The immunoprecipitates were analyzed for indicated proteins by western blot. (F) Huh7 cells were infected with JFH-1 (MOI = 0.1) after transfected with plasmids expressing SPSB2, SPSB2DSB or an empty vector. After 72 h, cells were exposed to 25 μg/ml cycloheximide (CHX) for the indicated periods. Whole cell lysates were then examined for the indicated proteins by western blot. (G) Huh7 cells were infected with JFH-1 (MOI = 0.1) after transfected with SiNC or SiSPSB2. After 72 h, cells were exposed to 25 μg/ml cycloheximide (CHX) for the indicated periods. Whole cell lysates were then examined for the indicated proteins by western blot.

Due to the vital role of SPSB2 in the ECS ubiquitin-proteasome system as a substrate adaptor resulting in protein degradation of the targets, the question of whether SPSB2 induces E1 and NS5A ubiquitination and degradation was explored. HEK293T cells were transfected with NS5A and ubiquitin in the presence of SPSB2 or SPSB2DSB, and the ubiquitination of NS5A was detected. When SPSB2 was not expressed, NS5A was ubiquitinated, indicating basal ubiquitination resulting from host proteins in 293T cells. However, SPSB2 expression resulted in a significantly greater level of NS5A ubiquitination than SPSB2DSB expression (Fig 5A). We also detected the effect of SPSB2 on E1 ubiquitination, and the results suggested that E1 was not ubiquitinated when SPSB2 was expressed (S4 Fig). To assess whether SPSB2 affects NS5A ubiquitination in an authentic infection system, Huh7 cells were infected with JFH-1 (MOI = 0.1) after transfected with the indicated plasmids. As shown in Fig 5D, a significantly greater level of NS5A ubiquitination was observed in SPSB2 expressing cells compared to cells transfected with mock vector or SPSB2DSB. While NS5A ubiquitination was reduced in SPSB2 knockdown cells compared to the control transfected with SiNC (Fig 5E). These results above indicated that SPSB2 promoted the ubiquitination of NS5A and was dependent on the SOCS box.

Next, to assess whether SPSB2 affects NS5A stability, the protein synthesis inhibitor cycloheximide was used. As shown in Fig 5B (left), in the absence of SPSB2, NS5A was relatively stable during the 6-h cycloheximide treatment. However, when SPSB2 was expressed, a marked decrease in the stability of NS5A was observed (Fig 5B, middle). Additionally, this effect was not observed when SPSB2DSB was expressed (Fig 5B, right). Also, to assess whether SPSB2 affects NS5A stability in an authentic infection system, Huh7 cells were infected with JFH-1 (MOI = 0.1) after transfected with the indicated plasmids. As shown in Fig 5F, a decrease in the stability of NS5A was observed in SPSB2 expressing cells compared to cells transfected with mock vector or SPSB2DSB during the 9-h cycloheximide treatment. While NS5A was relatively stable during the 9-h cycloheximide treatment in SPSB2 knockdown cells compared to the control transfected with SiNC(Fig 5G). These results above indicated that SPSB2 promoted the degradation of NS5A and was dependent on the SOCS box.

Finally, to investigate whether NS5A was degraded by SPSB2-mediated proteolysis, HEK293T cells were co-transfected with NS5A and SPSB2 and treated with the proteasome inhibitor MG132. The results showed that NS5A protein levels were decreased by SPSB2, and this reduction could be reversed by MG132 (Fig 5C). Collectively, these results suggested that SPSB2 mediates NS5A ubiquitination and degradation in a proteasome-dependent manner.

## Discussion

The ubiquitin-proteasome system plays a vital role in HCV replication and virus production. It has been reported that E6AP-mediated ubiquitination and degradation of the HCV core protein affects the production of HCV particles [29]. Zinc mesoporphyrin produces a rapid and profound downregulation of NS5A protein by enhancing its polyubiquitination and proteasome-dependent catabolism [30]. Additionally, TRIM14 and TRIM22 induce the ubiquitination and degradation of NS5A to inhibit HCV replication [15,16]. ISG12a recruits SKP2 for ubiquitination and degradation of viral protein NS5A to restrict viral infection [17]. hPLIC1 interacts with NS5B and decreases the NS5B protein level through ubiquitin-dependent degradation [31]. Fbw7 restricts the replication of HCV by targeting NS5B for ubiquitination and degradation [32]. In the present study, we first found that SPSB family proteins SPSB2 and SPSB3 inhibit HCV replication, in addition to their other roles in cellular immune response and cancer invasion and metastasis. SPSB2, the substrate-binding adaptor in the ECS system, interacts with the HCV protein NS5A via its SPRY domain and inhibits HCV replication by ubiquitination and degradation of NS5A.

HCV infection can affect the transcription and expression of host factors to regulate virus reproduction or cell survival. In the present study, SPSB family proteins SPSB2 and SPSB3 were upregulated after HCV infection, but SPSB1 and SPSB4 were not. The distinct mechanisms of change in SPSB proteins induced by HCV infection are still unknown and need for further study. Because all the expression levels of four SPSB proteins were low in hepatoma cells Huh7 and Huh7.5.1, we chose heterogenous overexpression to investigate the roles of SPSB proteins in HCV replication. We found that SPSB2 expression had the most prominent inhibitory effect on HCV replication among the SPSB proteins. Furthermore, this inhibition depended on SPSB2’s SOCS box binding to other proteins to form complexes in the ECS ubiquitin-proteasome system. This result indicated that increasing the expression of other proteins such as Elongin B/C or Cullin -5 may help SPSB2 inhibit HCV replication.

SPSB proteins include two functional domains: the SPRY domain and a SOCS box. The SPRY domain, which more than 150 human proteins contain, was discovered as a sequence repeat responsible for protein-protein interactions [33]. The SPRY domain of SPSB proteins interacts with many cellular proteins such as Par-4, c-Met, hnRNP A1, iNOS, TβRII, SNAIL, and EphB2[23–26,34–36]. In this study, we first identified viral proteins HCV E1 and NS5A as those interacting with the SPRY domain of SPSB2. E1 is an envelope glycoprotein that modulates HCV particle interaction with cellular receptor and participates in the assembly and release of viral particles during HCV morphogenesis [37]. However, we found that E1 was not the ubiquitination substrate in the SPSB2 ubiquitin-proteasome system. Whether the interaction between E1 and SPSB2 affects HCV entry and assembly and the release of viral particles should be investigated in future studies. Furthermore, these future studies could explore whether the other three members of SPSB proteins can interact with E1. We found that all four SPSB members can interact with NS5A but that the degree of NS5A ubiquitination was distinct (S5 and S6 Figs). These distinctions may be caused by different SPSBs sequences, protein structures, or binding affinity with NS5A. These distinctions may explain the different effects of SPSB family proteins on HCV RNA and protein levels when expressed in human hepatoma Huh7.5.1 cells (Fig 2A). Additionally, we discovered that SPSB2 targets NS5A for degradation; however, detailed functional domains or ubiquitinated sites are needed for this topic to be further explored. Besides viral proteins, SPSB2 may regulate other cellular factors participating in the HCV life cycle to inhibit HCV replication, the topic of which would be a good avenue for future study.

HCV infection is a major public health problem, and NS5A plays key roles in multiple aspects of the virus life cycle. In this study, we demonstrated that cellular factor SPSB2 interacts with and decreases viral protein NS5A to inhibit HCV replication. These findings add to our understanding of the interplay between HCV and host cells and provide a molecular basis for the potential development of novel therapeutics against HCV.

## Supporting information

S1 Fig Hepatitis C virus (HCV) RNA level was quantified by qRT-PCR. (A) Huh7.5.1 cells were incubated for 6 h with HCV JFH-1 (multiplicity of infection [MOI] = 1) and cultured for another 96 h with fresh medium; HCV RNA was quantified by qRT-PCR.(B) Huh7.5.1 cells were infected with JFH-1 (MOI = 1), incubated for 6 h, and harvested at different time points; HCV RNA was quantified by qRT-PCR. (C) Huh7.5.1 cells were infected with JFH-1 at different virus titers, incubated for 6 h, and harvested for HCV RNA detection after another 96 h by qRT-PCR. Experiments were performed three times with similar results.
(TIF)

S2 Fig The subcellular localization relationship between NS5A and SPSB2/mutants was determined in Huh7 cells. Huh7 cells were stained with anti-Myc antibody and TRITC-conjugated goat anti-rabbit secondary antibody to detect Myc-SPSB2 or its mutants, as well as stained with anti-Flag antibody and FITC-conjugated goat anti-mouse secondary antibody to detect Flag-NS5A after transfected with Flag-NS5A and Myc-SPSB2 or its mutants. Cells were stained with DAPI to visualize the nuclei.
(TIF)

S3 Fig The subcellular localization relationship between E1 and SPSB2/mutants was determined in Huh7 cells. Huh7 cells were stained with anti-Myc antibody and TRITC-conjugated goat anti-rabbit secondary antibody to detect Myc-SPSB2 or its mutants, as well as stained with anti-Flag antibody and FITC-conjugated goat anti-mouse secondary antibody to detect Flag-E1 after transfected with Flag-E1 and Myc-SPSB2 or its mutants. Cells were stained with DAPI to visualize the nuclei.
(TIF)

S4 Fig SPSB2 doesn’t induce the ubiquitination of Hepatitis C virus (HCV) E1. Flag-E1 and HA-Ub were transfected with Myc-SPSB2 or an empty vector in HEK293T cells for 48 h; cell lysates were immunoprecipitated with anti-Flag antibody, and the immunoprecipitates were analyzed for indicated proteins by western blot.
(TIF)

S5 Fig Hepatitis C virus (HCV) nonstructural protein 5A (NS5A) interacts with SPSB family proteins. HA-NS5A was co-transfected with Myc-tagged SPSB proteins (SPSB1,SPSB2, SPSB3, SPSB4) for 36 h in HEK293T cells; cell lysates were immunoprecipitated with IgG or anti-HA antibody, and the immunoprecipitates were analyzed by western blot with anti-HA or anti-Myc antibody.
(TIF)

S6 Fig SPSB family proteins induce the ubiquitination of nonstructural protein 5A (NS5A). Flag-NS5A and HA-Ub were transfected with Myc-tagged SPSB family proteins or an empty vector in HEK293T cells for 48 h; cell lysates were immunoprecipitated with anti-Flag antibody, and the immunoprecipitates were analyzed for indicated proteins by western blot.
(TIF)
