# Supplementary figures and images for: SPSB2 inhibits hepatitis C virus replication by targeting NS5A for ubiquitination and degradation
Source: PLoS One. 2019 Jul 25;14(7):e0219989. doi: 10.1371/journal.pone.0219989 (PMC6657855; doi:10.1371/journal.pone.0219989)

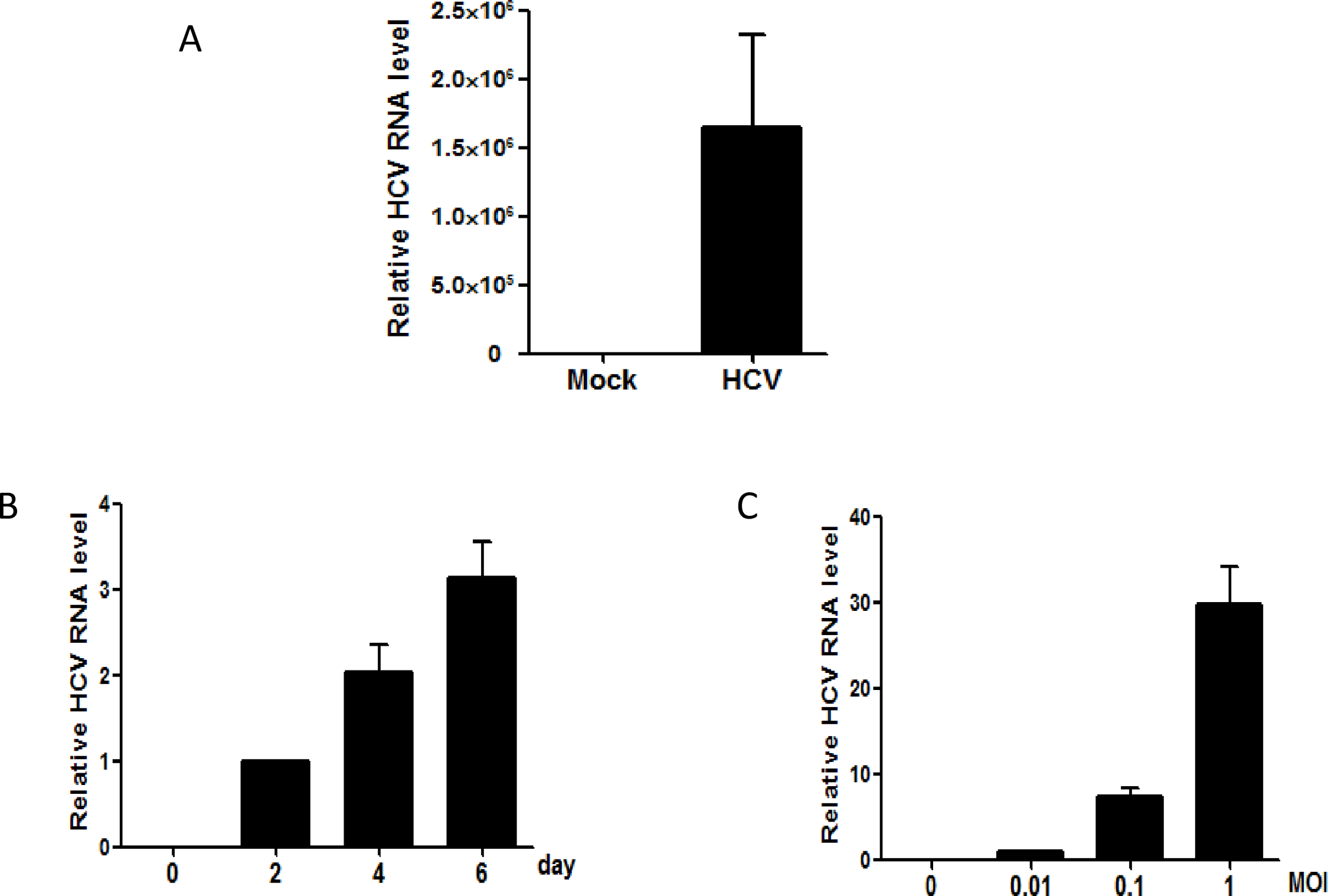

Supplement: S1 Fig — (A) Huh7.5.1 cells were incubated for 6 h with HCV JFH-1 (multiplicity of infection [MOI] = 1) and cultured for another 96 h with fresh medium; HCV RNA was quantified by qRT-PCR.(B) Huh7.5.1 cells were infected with JFH-1 (MOI = 1), incubated for 6 h, and harvested at different time points; HCV RNA was quantified by qRT-PCR. (C) Huh7.5.1 cells were infected with JFH-1 at different virus titers, incubated for 6 h, and harvested for HCV RNA detection after another 96 h by qRT-PCR. Experiments were performed three times with similar results. (TIF) [file pone.0219989.s001.tif]

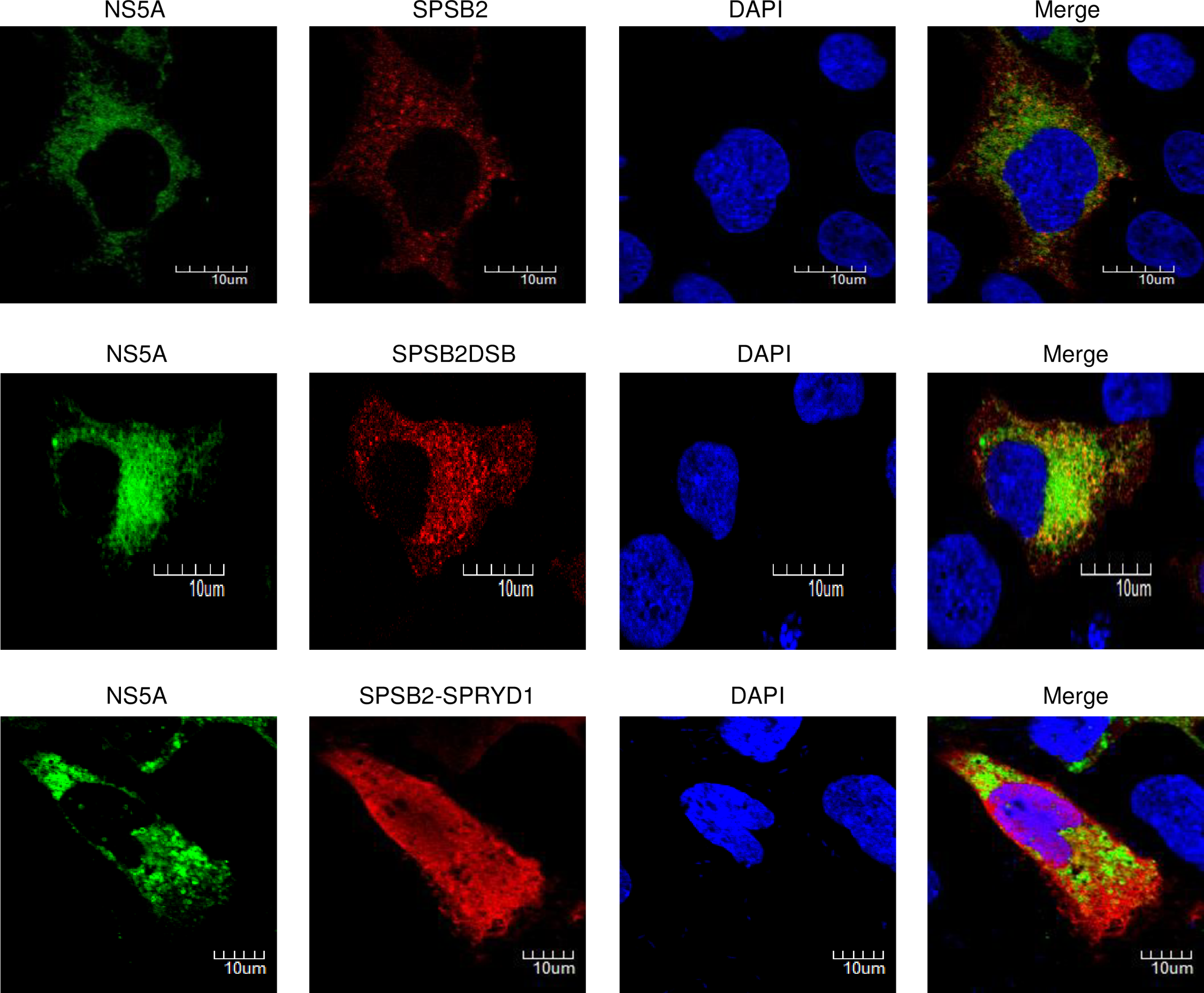

Supplement: S2 Fig — Huh7 cells were stained with anti-Myc antibody and TRITC-conjugated goat anti-rabbit secondary antibody to detect Myc-SPSB2 or its mutants, as well as stained with anti-Flag antibody and FITC-conjugated goat anti-mouse secondary antibody to detect Flag-NS5A after transfected with Flag-NS5A and Myc-SPSB2 or its mutants. Cells were stained with DAPI to visualize the nuclei. (TIF) [file pone.0219989.s002.tif]

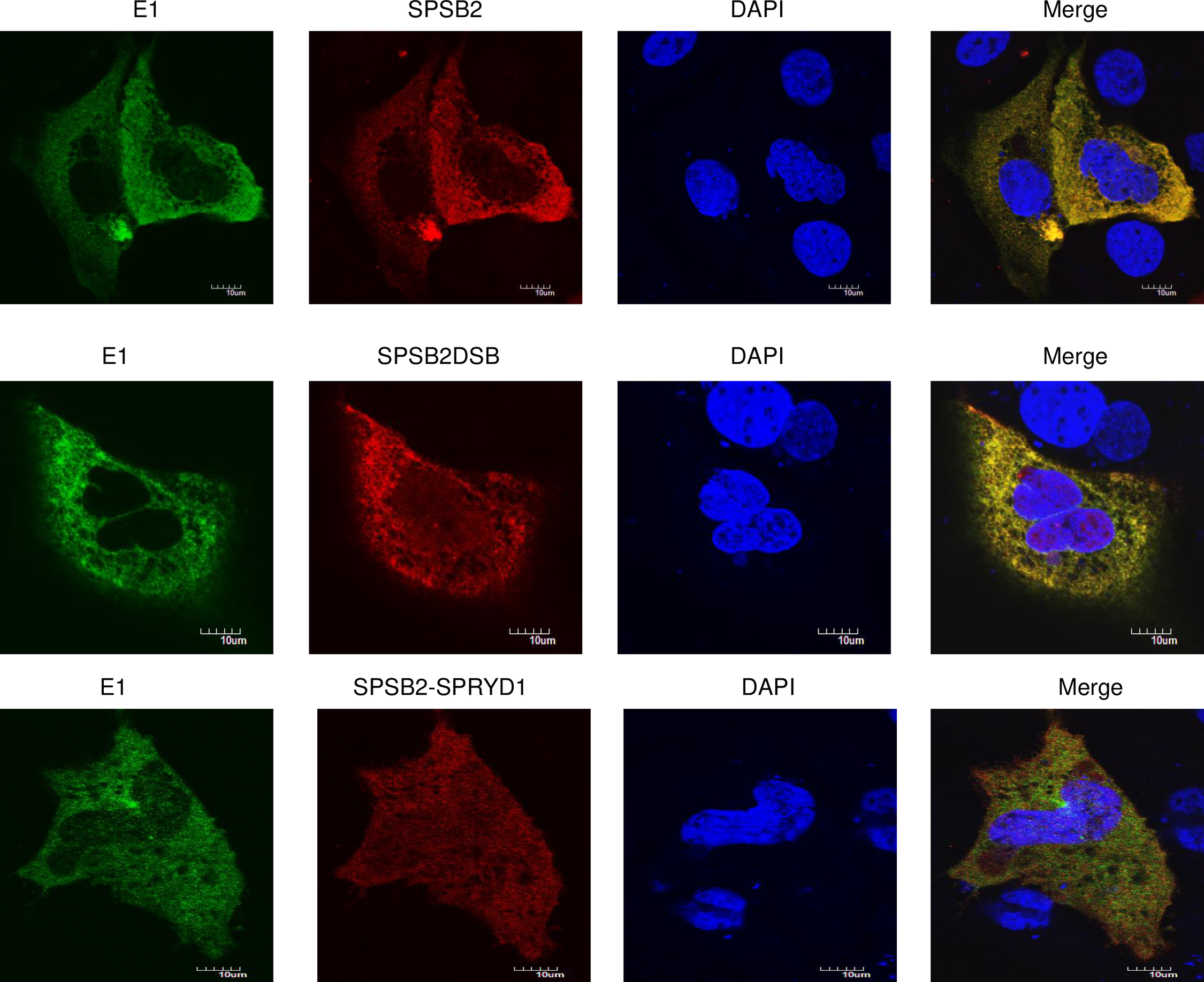

Supplement: S3 Fig — Huh7 cells were stained with anti-Myc antibody and TRITC-conjugated goat anti-rabbit secondary antibody to detect Myc-SPSB2 or its mutants, as well as stained with anti-Flag antibody and FITC-conjugated goat anti-mouse secondary antibody to detect Flag-E1 after transfected with Flag-E1 and Myc-SPSB2 or its mutants. Cells were stained with DAPI to visualize the nuclei. (TIF) [file pone.0219989.s003.tif]

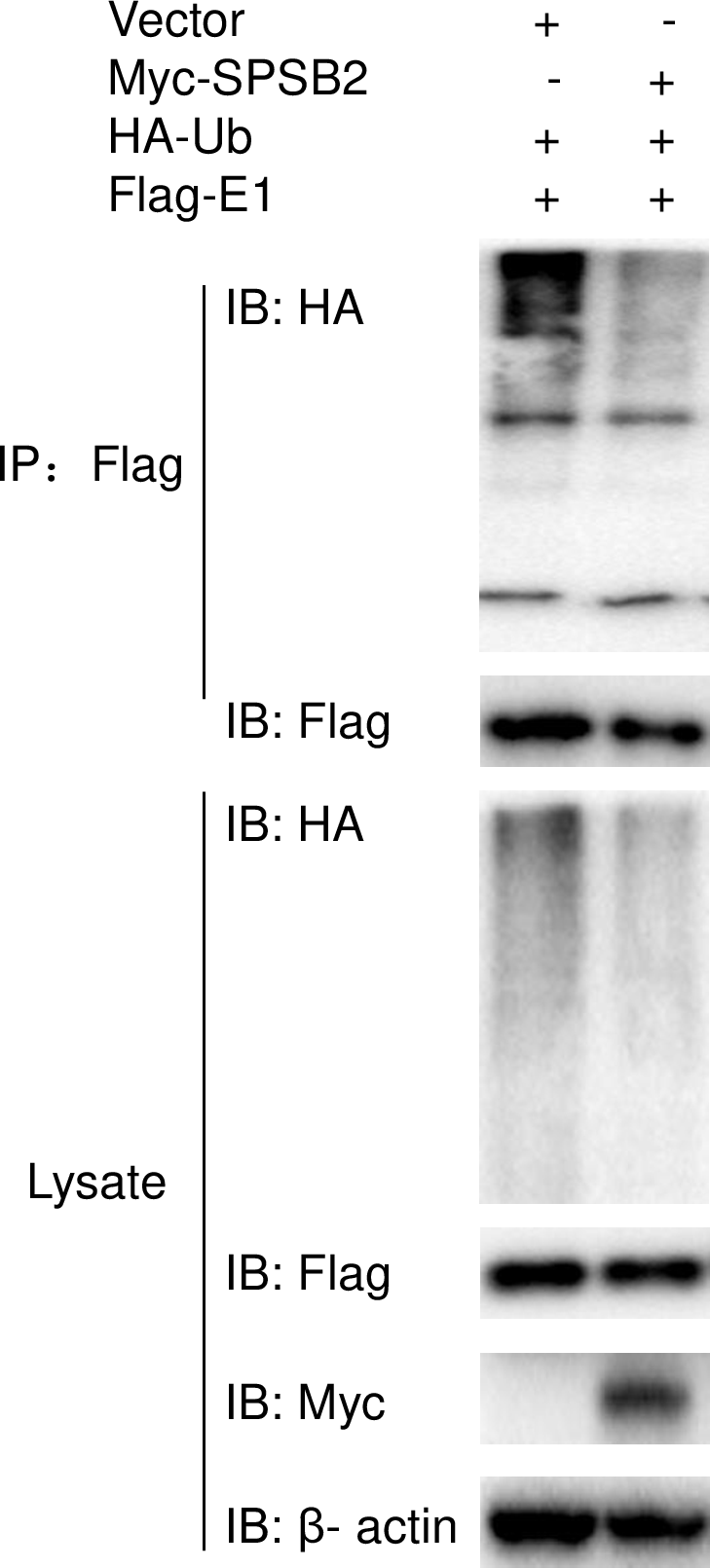

Supplement: S4 Fig — Flag-E1 and HA-Ub were transfected with Myc-SPSB2 or an empty vector in HEK293T cells for 48 h; cell lysates were immunoprecipitated with anti-Flag antibody, and the immunoprecipitates were analyzed for indicated proteins by western blot. (TIF) [file pone.0219989.s004.tif]

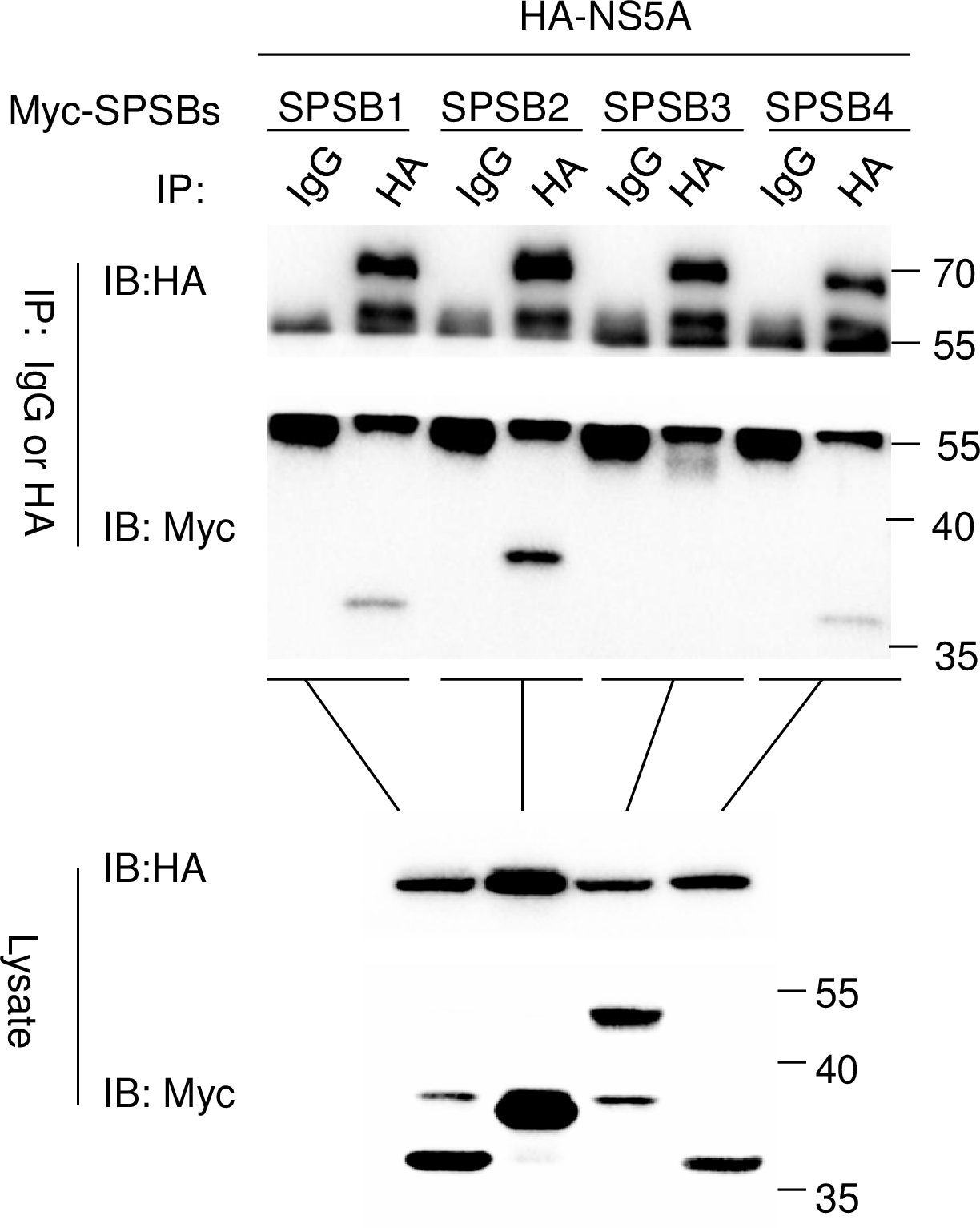

Supplement: S5 Fig — HA-NS5A was co-transfected with Myc-tagged SPSB proteins (SPSB1,SPSB2, SPSB3, SPSB4) for 36 h in HEK293T cells; cell lysates were immunoprecipitated with IgG or anti-HA antibody, and the immunoprecipitates were analyzed by western blot with anti-HA or anti-Myc antibody. (TIF) [file pone.0219989.s005.tif]

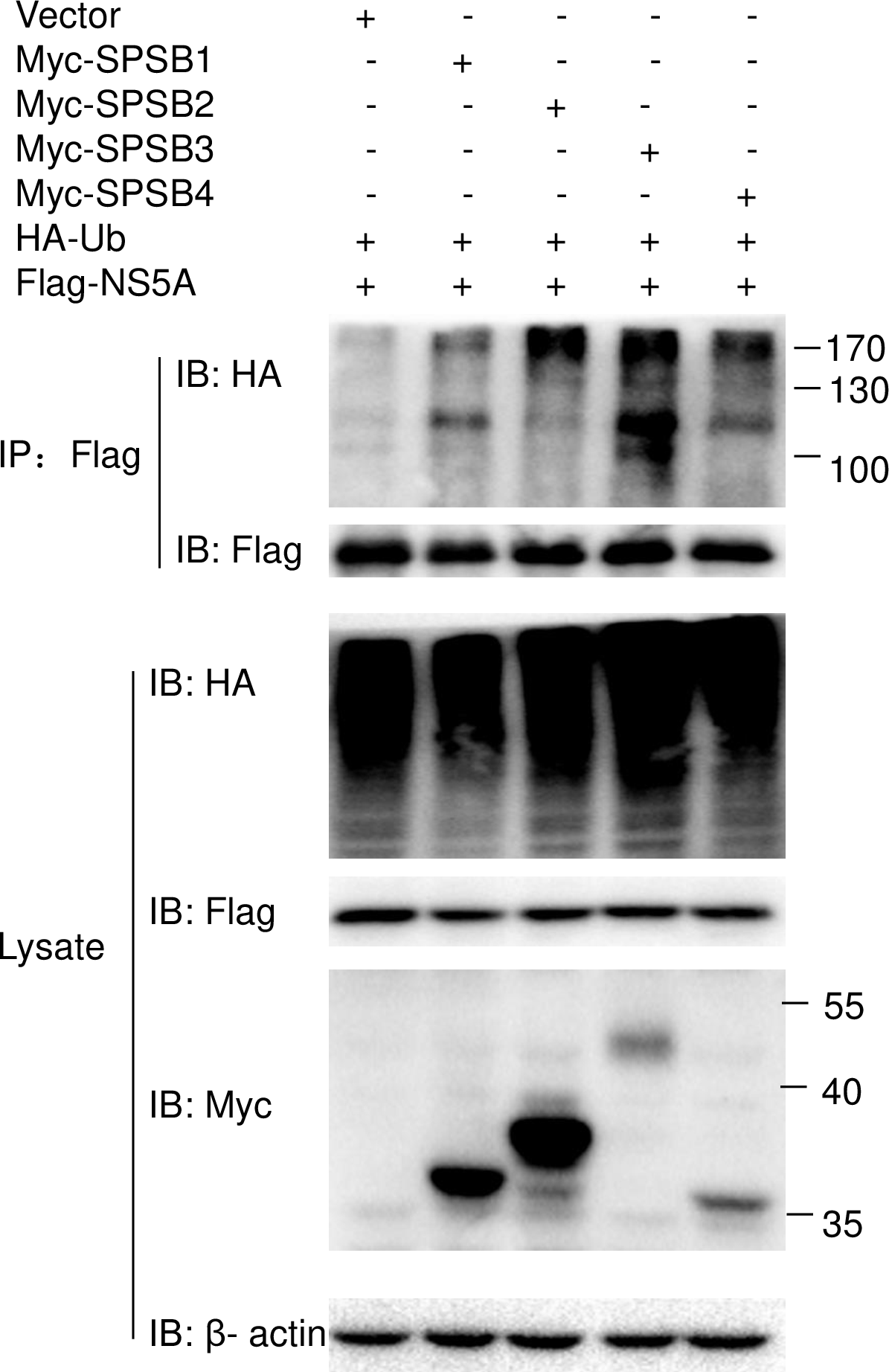

Supplement: S6 Fig — Flag-NS5A and HA-Ub were transfected with Myc-tagged SPSB family proteins or an empty vector in HEK293T cells for 48 h; cell lysates were immunoprecipitated with anti-Flag antibody, and the immunoprecipitates were analyzed for indicated proteins by western blot. (TIF) [file pone.0219989.s006.tif]
